# The Evaluation of Mini-Clinical Evaluation Exercise (Mini-CEX) for Assessing Neurology Cases Among Postgraduate Medicine Students

**DOI:** 10.7759/cureus.73641

**Published:** 2024-11-13

**Authors:** Afreen Khan, Rosmy Jose, Azhar Uddin, Sajad Ul Islam

**Affiliations:** 1 Internal Medicine, Hamdard Institute of Medical Sciences and Research, New Delhi, IND; 2 Community Medicine, Hamdard Institute of Medical Sciences and Research, New Delhi, IND

**Keywords:** formative assessment, medical education, mini-cex, neurology, workplace-based assessment

## Abstract

Background

Traditional assessments in postgraduate medical training tend to emphasize cognitive skills while often neglecting the psychomotor and affective domains. The mini-clinical evaluation exercise (mini-CEX) addresses these limitations by evaluating clinical, communication, and humanistic skills with immediate feedback. This study aimed to evaluate the acceptability and feasibility of the mini-CEX in assessing neurology cases among postgraduate internal medicine students at a medical college in North India.

Methods

Between March and July 2024, 10 postgraduate medicine students and 14 faculty members participated in the study. Each student completed at least four mini-CEX sessions, during which performance across seven core skills was rated using a nine-point scale. Immediate feedback was provided by faculty following each session. Both student and faculty feedback were collected to assess the acceptability and feasibility of the mini-CEX. Descriptive statistics were used to evaluate these factors, while paired t-tests were applied to measure improvements in student performance.

Results

A total of 63 mini-CEX sessions were conducted, with an average observation time of 18.77 minutes and an average feedback time of 10.08 minutes. Significant improvements in clinical skills were observed among second-year (P = 0.04) and third-year (P = 0.01) residents, with second-year residents also showing gains in humanistic skills (P = 0.03). Student feedback was largely positive, with nine out of 10 (90%) participants expressing satisfaction and eight out of 10 (80%) recommending the continued use of the tool. Faculty feedback indicated that 10 of 14 (71.4%) supported its continued use and eight of 14 (57%) found it better than traditional assessment methods.

Conclusions

The mini-CEX is a feasible and acceptable formative assessment tool for neurology cases, effectively enhancing clinical and communication skills. Despite concerns regarding subjectivity, the mini-CEX shows promise for wider implementation in postgraduate, and potentially undergraduate, medical training.

## Introduction

The assessment of clinical skills in medical education is a crucial aspect of postgraduate training worldwide. Traditional methods, such as long and short case presentations and viva voce examinations, are commonly utilized across various medical specialties, including internal medicine. These assessments often encompass multiple systems, such as neurology, cardiology, and respiratory systems. Neurology cases, in particular, are predominantly evaluated through long case presentations and viva voce during both summative and formative assessments. While these methods emphasize presentation skills and cognitive aspects, they tend to underemphasize the evaluation of psychomotor skills and largely neglect the affective domain, including crucial communication skills [1]. Moreover, these traditional approaches often lack the direct observation of clinical encounters, and the feedback provided is typically unstructured.

Over the past three decades, workplace-based assessment (WPBA) has been introduced globally in postgraduate training to address these limitations. Several factors contribute to the effectiveness of WPBA, including feedback tailored to the learner's needs and focused on essential performance aspects [2]. One such WPBA method is the mini-clinical evaluation exercise (mini-CEX). The mini-CEX involves a faculty member observing and assessing a resident as they perform a focused history and physical examination in various settings, such as the inpatient department (IPD), outpatient department (OPD), or emergency department. Following the clinical encounter, the resident provides a diagnosis and treatment plan, which is evaluated by the faculty member, who also provides structured educational feedback. These sessions are brief, allowing for multiple assessments of each resident by different faculty members. The original mini-CEX uses a nine-point rating scale divided into three categories: unsatisfactory (1-3), satisfactory (4-6), and superior (7-9) [3].

The integration of the mini-CEX for formative assessment in postgraduate medical training is particularly useful for improving clinical and counseling skills. The structured feedback offered through this tool helps students refine their decision-making processes and enhances overall skill development. Although the mini-CEX is widely implemented in Western countries, its adoption in Indian medical colleges has been limited [4]. By incorporating the mini-CEX into existing assessment frameworks, gaps in the comprehensive evaluation of clinical competencies, including communication and psychomotor skills, can be bridged. This study aims to assess the acceptability and feasibility of using the mini-CEX for evaluating clinical, counseling, and professional skills in neurology cases among postgraduate medicine students.

## Materials and methods

Study design

This was a single-center, prospective interventional study conducted in the Department of Medicine at a medical college in North India. The objective was to evaluate the acceptability and feasibility of the mini-clinical evaluation exercise (mini-CEX) as a formative assessment tool for postgraduate internal medicine students. The study was carried out over a period of five months, from March 2024 to July 2024.

Study participants

Ten postgraduate students from the Department of Internal Medicine participated in the study. The selection of participants was based on convenience sampling, taking into consideration their availability and willingness to engage in the study. Fourteen faculty members from the same department, each with at least five years of teaching experience, served as assessors during the mini-CEX sessions.

Study procedure

Both postgraduate students and faculty members attended sensitization sessions that explained the concept and implementation of the mini-CEX. The faculty participated in a two-hour workshop that detailed the objectives, structure, and scoring system of the mini-CEX, with a special focus on the importance of delivering constructive, structured, and immediate feedback. Postgraduate students attended a one-hour orientation session that introduced them to the format, objectives, and integration of the mini-CEX into their clinical training.

Each student underwent a minimum of four mini-CEX assessments, conducted in either inpatient or outpatient departments. Neurology cases were selected to ensure consistency in case difficulty and to align with the postgraduate curriculum for internal medicine training.

Mini-CEX assessment tool

The mini-CEX assessment tool, developed by the American Board of Internal Medicine, was employed to evaluate seven core clinical skills. These skills, medical interviewing, physical examination, clinical judgment, professionalism/humanistic qualities, counseling, organization, and overall clinical competence, were assessed using a nine-point Likert scale. Scores from 1 to 3 were categorized as unsatisfactory, scores from 4 to 6 as satisfactory, and scores from 7 to 9 as superior.

Assessments took place in real time, either during ward rounds (inpatient settings) or patient consultations (outpatient settings). Each mini-CEX session was designed to last between 15 and 20 minutes, followed by an additional 5-10 minutes for immediate feedback provided by the assessing faculty member.

Data collection

Performance scores were documented using the standardized mini-CEX pro forma following each session. The duration of both the observation and feedback segments was also recorded. After the completion of all sessions, both students and faculty provided feedback using a custom-designed questionnaire. These questionnaires, developed through expert consensus and peer-reviewed for content validation, included Likert-scale items (ranging from 1 to 5, where 1 indicated strong disagreement and 5 indicated strong agreement) and open-ended sections for suggestions and comments (Appendices). The feedback focused on the participants' perceptions of the mini-CEX's acceptability, feasibility, and effectiveness as an assessment tool.

Statistical analysis

Data were analyzed using SPSS software (version 26) (IBM Corp., Armonk, NY). Descriptive statistics, including mean, standard deviation, frequencies, and percentages, were employed to summarize the data. To assess improvement in performance over time, paired t-tests were used to compare the scores from the first and last mini-CEX sessions in both clinical skills and humanistic/counseling skills. A p-value of less than 0.05 was considered indicative of statistical significance.

Feedback form responses were analyzed quantitatively by compiling the frequency and percentage of each rating. Qualitative data, including comments and suggestions, were reviewed and summarized to highlight recurring themes and areas for improvement. This combined quantitative and qualitative analysis provided a comprehensive understanding of the participants' perspectives on the mini-CEX.

## Results

A total of 10 postgraduate students participated in the study, comprising three first-year, three second-year, and four third-year residents. Across these participants, 63 mini-CEX assessments were completed, with each student undergoing between four and nine sessions. Specifically, first-year residents participated in four evaluations each, second-year residents in 6-8 evaluations, and third-year residents in 6-9 evaluations. Fourteen faculty members conducted these assessments, averaging approximately five mini-CEX sessions per faculty member over the four-month study period, equating to 1.2 sessions per month per faculty member.

The mean observation time per session was 18.77 ± 4.75 minutes, with an average feedback duration of 10.08 ± 5.20 minutes. The majority of the neurology cases were drawn from the inpatient department (95.2%), with a smaller proportion from the outpatient department (4.7%). In terms of case complexity, most cases were categorized as moderately complex (79.3%, n = 50), while low-complexity cases were less common (12.6%, n = 8), and high-complexity cases were rare (7.9%, n = 5). The complexity of each case was determined by the evaluating faculty member.

Mini-CEX scores

The mini-CEX scores were analyzed, with a year-wise comparison conducted for key clinical skills such as medical interviewing, physical examination, clinical judgment, and overall clinical competence. The results of these comparisons are summarized in Table 1.

**Table 1:** First versus last mini-CEX scores of clinical skills mini-CEX, mini-clinical evaluation exercise; SD, standard deviation

Residency year	First mini-CEX score, total marks -36 (mean ± SD)	Last mini-CEX score, total marks -36 (mean ± SD)	P-value
First	19.66 ± 6.35	24 ± 1	0.363
Second	16.3 ± 4.50	24.6 ± 1.52	0.042
Third	19 ± 2.58	24.5 ± 1.73	0.019

Similarly, a year-wise comparison of scores for humanistic and counseling skills was conducted, as summarized in Table 2.

**Table 2:** First versus last mini-CEX scores of humanistic and counseling skills mini-CEX, mini-clinical evaluation exercise; SD, standard deviation

Residency year	First mini-CEX score, total marks -18 (mean ± SD)	Last mini-CEX score, total marks -18 (mean ± SD)	P-value
First	9.66 ± 2.88	12.66 ± 1.15	0.095
Second	9 ± 2	13.66 ± 2.08	0.033
Third	10.75 ± 2.98	13.25 ± 0.95	0.228

Residents exhibited improvement in both clinical and humanistic/counseling skills between the first and last mini-CEX assessments. A paired t-test was conducted to compare the initial and final scores for each resident cohort (first, second, and third years). Second-year residents demonstrated significant improvement in both clinical skills (P = 0.042) and humanistic/counseling skills (P = 0.033). Third-year residents also showed a statistically significant improvement in clinical skills (P = 0.019). Although first-year residents displayed progress in both skill areas, the changes were not statistically significant.

Student feedback

**Figure 1:**
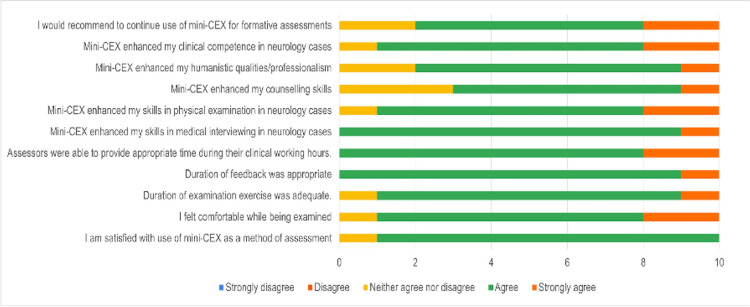
Student feedback mini-CEX: mini-clinical evaluation exercise

Student feedback is illustrated in Figure 1.

The students reported a high level of satisfaction with the mini-CEX as a formative assessment tool. Ninety percent (nine out of 10) of the students rated their satisfaction as 4 on a five-point Likert scale. Notably, students acknowledged the positive impact of mini-CEX on both their clinical and humanistic skills. Ninety percent (nine out of 10) of the participants agreed that the mini-CEX improved their medical interviewing and physical examination skills. In terms of counseling skills, 60% (six out of 10) reported improvement, while 30% (three out of 10) remained neutral. Additionally, 90% (nine out of 10) of students felt that their humanistic skills were enhanced, with 20% (two out of 10) strongly agreeing.

Students also expressed comfort during the assessment process, with a mean comfort score of 4.1 out of 5. The duration of the assessment and feedback was deemed appropriate, receiving mean scores of 4.0 and 4.1, respectively. Furthermore, 80% (eight out of 10) of the students recommended the continued use of the mini-CEX for formative assessments.

Faculty feedback

**Figure 2:**
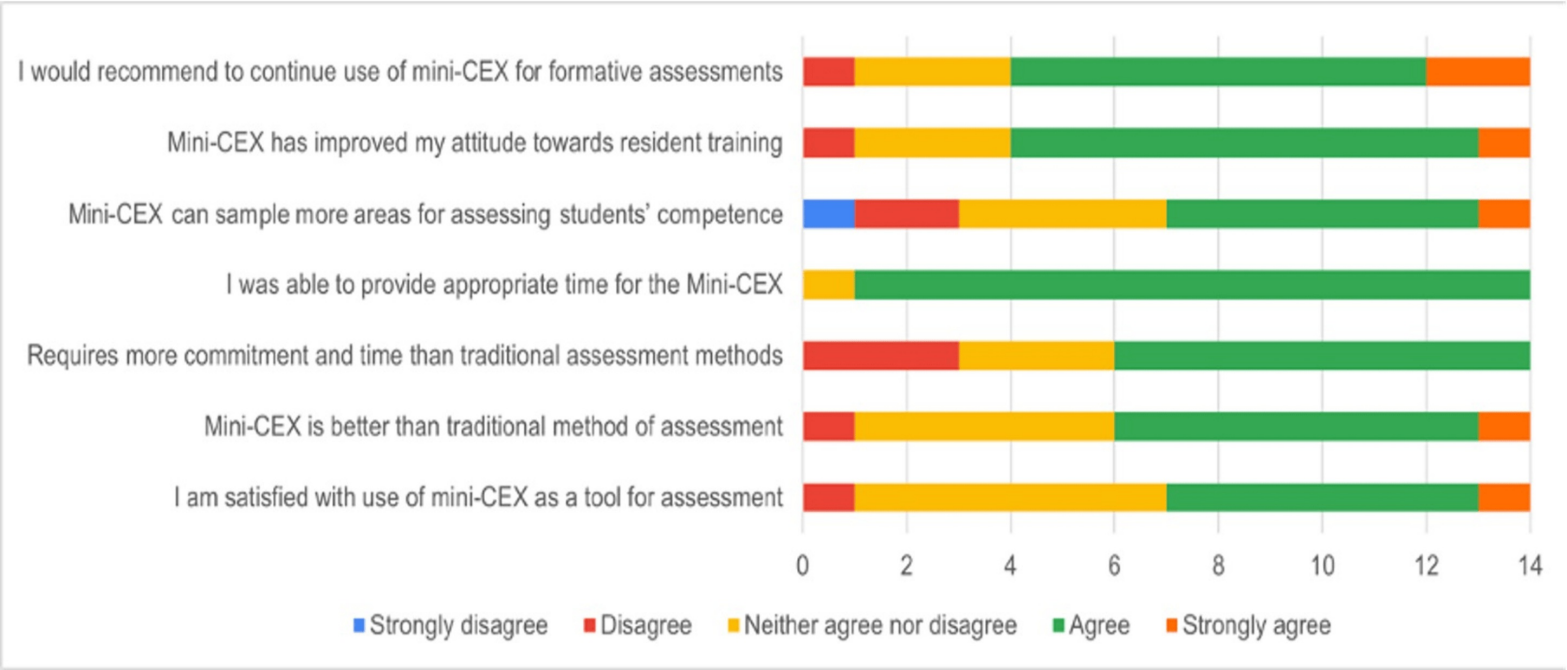
Faculty feedback mini-CEX: mini-clinical evaluation exercise

Faculty feedback is illustrated in Figure 2.

Out of the 14 participating faculty members, 71.4% (10 out of 14) indicated that they would recommend the continued use of the mini-CEX for formative assessments. Half of the faculty (50%, seven out of 14) rated their satisfaction with the mini-CEX at 4 or higher on a five-point Likert scale, while 42.8% (six out of 14) reported neutral satisfaction with a score of 3. Additionally, 57% (eight out of 14) of the faculty perceived the mini-CEX as a superior assessment tool when compared to traditional methods.

Furthermore, the majority of faculty (92.8%, 13 out of 14) affirmed that they were able to allocate sufficient time for conducting mini-CEX sessions. The tool was noted to improve faculty attitudes toward resident training, with a mean score of 3.71 in this regard. However, faculty members highlighted the subjectivity inherent in the tool and suggested the need for pre-evaluation calibrations to better define the criteria for unsatisfactory, satisfactory, and superior ratings. They also recommended the more frequent use of the mini-CEX to improve familiarity with the tool.

## Discussion

The mini-CEX has gained widespread adoption across various medical disciplines as a formative assessment tool for both undergraduate and postgraduate students. However, its use in assessing neurology cases among postgraduate medicine students remains relatively underexplored. This study addresses this gap by evaluating the application of mini-CEX in neurology cases, aiming to assess its feasibility and acceptability in this context.

Our findings suggest that implementing the mini-CEX is feasible within existing clinical environments, without significantly burdening faculty or resources. Each faculty member conducted an average of 1.2 mini-CEX sessions per month, indicating that the tool was manageable and did not increase the workload substantially. This aligns with a similar study conducted among postgraduate surgery students, where faculty performed one mini-CEX session per month [4]. The mean observation time was 18.77 minutes, and the mean feedback time was 10.08 minutes, resulting in an average total evaluation time of 28.77 minutes. These results are comparable to findings from a study by Wiles et al., where the average time for neurology evaluations, including feedback, was approximately 25 minutes [5]. The slightly longer duration of our study may be attributed to the complexity of neurology cases or the relative inexperience of faculty with the mini-CEX, as multiple skills were often assessed concurrently. This challenge can be addressed through faculty development workshops focusing on workplace-based assessment (WPBA) and the consistent use of mini-CEX, with an emphasis on specific skills to enhance feasibility. Notably, the majority of faculty members (92.8%) reported being able to allocate adequate time for mini-CEX sessions. This is comparable to an emergency department study where 70.6% of assessors could appropriately integrate mini-CEX within their clinical shifts [6].

The majority of neurology cases (95.2%) were derived from the IPD, with a smaller proportion (4.7%) from the OPD. This distribution likely reflects the extended interaction time available in the IPD compared to the more fast-paced OPD environment. In contrast, Batra et al. reported a more balanced case distribution, with 50% of sessions conducted with ambulatory patients, 43.75% with inpatients, and 6.25% in the emergency department [7]. Similarly, Singh and Sharma reported that 75% of sessions were conducted in the OPD and 25% in the IPD [8].

Significant improvements in clinical skills were observed among second- and third-year residents, which is consistent with findings from Wiles et al., who also reported an increase in trainee competence and communication skills over time [5]. While this could reflect a sampling bias, the increasing scores with extended training and additional evaluations provide compelling evidence supporting the validity of the mini-CEX. Additionally, significant improvements were observed in humanistic and counseling skills, particularly among second-year residents, a finding that mirrors results from earlier studies [7,9-11]. These studies have highlighted the effectiveness of the mini-CEX in fostering competencies such as communication and professionalism.

The high level of student satisfaction further supports the acceptability of the mini-CEX. Ninety percent of students rated their satisfaction as 4 out of 5 on a Likert scale, demonstrating the tool's ability to meet their expectations and educational needs. This finding is in line with previous research on the acceptability of mini-CEX in postgraduate settings [4,12,13]. Structured feedback, one of the most valued aspects of the mini-CEX, received a mean score of 4.1 for appropriateness, with the overall session duration also rated positively at 4.0. These findings are comparable to a study by Gupta et al., where the feedback duration was similarly rated at 4.1 [12]. Moreover, 80% of participating students recommended the continued use of mini-CEX for formative assessment, a recommendation echoed in studies involving anesthesia postgraduate students, where over 90% endorsed its ongoing use [13].

Faculty feedback also underscored the acceptability of the mini-CEX, with 71.4% recommending its continued use and 57% recognizing it as a superior assessment tool compared to traditional methods. These findings are consistent with regional data [4,7,12]. A concern raised by faculty was the subjectivity of the mini-CEX, with suggestions for pre-evaluation calibration to better delineate unsatisfactory, satisfactory, and superior scores. While inter-rater reliability remains a challenge for both traditional assessments and the mini-CEX, the brevity of the mini-CEX allows for multiple observations, collectively ensuring acceptable reliability [14]. The quality of the mini-CEX, as well as participant responsiveness, is crucial to maximizing its educational impact, underscoring the importance of its proper implementation [15]. Previous research has confirmed the reliability of mini-CEX in assessing clinical skills, making it a valuable tool in the evaluation of medical trainees [16]. As WPBA continues to gain traction, there is a growing need for ongoing faculty development and stronger evidence supporting the validity and reliability of these tools. This will enable academic institutions to effectively integrate mini-CEX into their existing curricula [17].

Limitations

Despite the generally high acceptability of the mini-CEX, several challenges were identified. As this study was conducted within a single specialty and institution, the findings may not be generalizable to residents in other settings or contexts. A notable concern raised by faculty members was the subjective nature of the tool, which could result in inconsistent evaluations and potentially compromise the reliability of the assessments. Additionally, there was variability in the number of mini-CEX evaluations per resident, ranging from four to nine sessions. This variation could have influenced the reliability of the observed improvements in scores, as a greater number of evaluations might provide a more accurate representation of each resident's progress.

## Conclusions

The acceptability and feasibility of the mini-CEX as a formative assessment tool for postgraduate internal medicine students are strongly supported by both student and faculty feedback. Its ability to deliver structured and meaningful feedback, alongside its positive impact on the development of clinical and humanistic skills, is a pivotal factor contributing to its widespread acceptance. Addressing the challenge of subjectivity through strategies such as pre-evaluation calibration and the more frequent use of the tool could further improve its reliability. Overall, the mini-CEX shows great potential for enhancing the quality of medical education and merits broader implementation, alongside further research to optimize its use across various medical specializations.

The successful application of the mini-CEX in neurology cases suggests its potential for broader use in other internal medicine subspecialties, such as respiratory and cardiovascular systems. Expanding its adoption to undergraduate students could also be considered to further improve the assessment of their clinical skills.

## Appendices

Table 3 and Table 4 show the student and faculty feedback questionnaires, respectively.

**Table 3:** Student feedback questionnaire mini-CEX: mini-clinical evaluation exercise

Serial number	Statement	Strongly disagree (1)	Disagree (2)	Neither agree nor disagree (3)	Agree (4)	Strongly agree (5)
1	I am satisfied with the use of mini-CEX as a method of assessment					
2	I felt comfortable while being examined					
3	Duration of the examination exercise was adequate					
4	Duration of feedback was appropriate					
5	Assessors were able to provide appropriate time during their clinical working hours					
6	Mini-CEX enhanced my skills in medical interviewing in neurology cases					
7	Mini-CEX enhanced my skills in physical examination in neurology cases					
8	Mini-CEX enhanced my counseling skills					
9	Mini-CEX improved my professionalism					
10	Mini-CEX enhanced my clinical competence in neurology cases					
11	I would recommend to continue the use of mini-CEX for formative assessments					
Suggestions/comments						

**Table 4:** Faculty feedback questionnaire mini-CEX: mini-clinical evaluation exercise

Serial number	Statement	Strongly disagree (1)	Disagree (2)	Neither agree nor disagree (3)	Agree (4)	Strongly agree (5)
1	I am satisfied with the use of mini-CEX as a tool for assessment					
2	Mini-CEX is better than traditional method of assessment					
3	Requires more commitment and time than traditional assessment methods					
4	I was able to provide appropriate time for the mini-CEX					
5	Mini-CEX can sample more areas for assessing students' competence					
6	Mini-CEX has improved my attitude toward resident training					
7	I would recommend to continue the use of mini-CEX for formative assessments					
Suggestions/comments						
